# A Combinational Strategy for Effective Heterologous Production of Functional Human Lysozyme in *Pichia pastoris*

**DOI:** 10.3389/fbioe.2020.00118

**Published:** 2020-03-10

**Authors:** Huahua He, Shijie Wu, Meng Mei, Jiali Ning, Chaoyin Li, Lixin Ma, Guimin Zhang, Li Yi

**Affiliations:** State Key Laboratory of Biocatalysis and Enzyme Engineering, Hubei Engineering Research Center for Bio-enzyme Catalysis, Hubei Key Laboratory of Industrial Biotechnology, College of Life Sciences, Hubei University, Wuhan, China

**Keywords:** human lysozyme, antimicrobial agent, heterologous expression, molecular chaperones, *P. pastoris*, high-density cultivation

## Abstract

Human lysozyme (hLYZ), known for its bacteriolytic activity, is widely applied in the food and pharmaceutical industries as an antimicrobial agent. However, its extensive application was limited by its low large-scale production efficiency. In this study, a combinational method of integrating codon optimization, multiple gene copies, and ER molecular chaperone co-expression was developed to improve the heterologous production of hLYZ in *Pichia pastoris* GS115. Our results showed that increasing the copy number of the optimized hLYZ gene in *P. pastoris* could enhance its secretory production level up to 1.57-fold. The recombinant opt-hLYZ-6C strain that contains six copies of opt-hLYZ gene exhibited the highest mRNA transcription levels, giving the highest production of 0.22 ± 0.02 mg/mL of hLYZ in the medium supernatant with a bacteriolytic activity of 14,680 ± 300 U/mL against *Micrococcus lysodeikticus* in the shaking flask experiment. Moreover, co-overexpression of ER retention molecular chaperones, such as Pdi1 or Ero1, in the recombinant opt-hLYZ-6C strain both presented positive effects on the secretory production of hLYZ. Our further characterization indicated that tandem co-expression of Ero1 and Pdi1 together presented an added-up effect. The secretory production of hLYZ in the medium supernatant reached 0.34 ± 0.02 mg/mL of the recombinant opt-hLYZ-6C-EP strain in the shaking flask experiment, with a bacteriolytic activity of 21,200 ± 400 U/mL. Compared to the recombinant opt-hLYZ-1C strain, these final improvements were calculated as 2.43-fold and 2.30-fold on secretory protein levels and antibacterial activity, respectively. Finally, the recombinant opt-hLYZ-6C-EP strain was applied for high-density cultivation in 5 L of fermenter, in which the secretory yield of hLYZ reached 2.34 ± 0.02 mg/mL in the medium supernatant, with a bacteriolytic activity of 1.76 ± 0.02 × 10^5^ U/mL against *M. lysodeikticus*. All these numbers presented the highest heterologous production levels of hLYZ in microbial systems.

## Introduction

Lysozyme (EC 3.2.1.17) belongs to ovo-antimicrobials, which has a well-characterized bacteriolytic characteristic mainly against Gram + bacteria, such as *Lactobacillus brevis*, *Micrococcus lysodeikticus*, and *Pediococcus damnosus* (Ercan and Demirci, 2016). Lysozyme is widely distributed in the tissues of humans, animals, plants, and even certain microorganisms (Ogawa et al., 1971), functioning by destroying bacterial cell wall through cleaving the chemical bonds linking the N-acetylglucosamine and N-acetylmuramic acid in the peptidoglycan layer (Jollès and Jollès, 1984; Johnson, 1998; Vocadlo et al., 2001). As one of the first solved protein structures, the crystal structure of lysozyme (chicken source) was resolved in 1965 (Blake et al., 1965; Johnson and Phillips, 1965), which laid a good foundation on its biochemical analysis. Till so far, different kinds of lysozymes are classified into six major types, depending on their molecular structure, source, and molecular weight (Weaver et al., 1984). Among them, the c-type lysozymes from chicken and human lysozyme were mostly studied, due to their good antibacterial properties and serving as models for enzyme structure and function studies (Peters et al., 1989; Prager and Jollès, 1996; Callewaert and Michiels, 2010).

Lysozyme possesses properties for bacterial killing and inflammation elimination, and also is found to prompt virus inactivation (Ferrari et al., 1959; Khalil et al., 1989). It has been used to replace antibiotics as food additives to inhibit bacterial growth, feed additives to prevent feed mildew, and also as anti-infection agent in pharmaceutics (Jollès and Jollès, 1984; Hughey et al., 1989; Biswas et al., 2016). Chicken lysozyme is widely used, while its bacteriolytic activity is almost four times lower than that of human lysozyme (hLYZ) (Ercan and Demirci, 2016). In addition, there are reports that hLYZ has better thermo-stability (Li et al., 1995), and it is safer and less antigenic than chicken lysozyme, especially for use in human food and therapeutics (Morita et al., 1995; Ercan and Demirci, 2016). Although hLYZ has many advantages in replacing antibiotics, its large-scale production is very challenging. Currently, hLYZ is mainly extracted from human milk and placenta, which is restricted by lack of raw materials and high cost of purification (Wei et al., 2012). Hence, it is necessary to develop heterologous production strategies of hLYZ in microbial systems to meet market demands.

Since hLYZ is a eukaryotic protein containing four intramolecular disulfide bonds, its expression in *Escherichia coli* cells are not favored. Most successful efforts for heterologous production of hLYZ that have been made so far exist in fungus, including intracellular expression in *Saccharomyces cerevisiae* (Choi et al., 2004), and extracellular secretory production in *P. pastoris* (Wei et al., 2012; Yu et al., 2014), *Kluyveromyces lactis* K7 (Ercan and Demirci, 2015), and *Aspergillus oryzae* (Jin et al., 2016). Among these microbial hosts, *P. pastoris*, with strong protein secretion ability and well-developed high-density cultivation techniques, is an ideal host for the heterologous production of hLYZ for industrial needs. Moreover, *P. pastoris* is also identified as a GRAS strain, which favors its potential applications in food and therapeutics. Several attempts of expressing chicken or human lysozymes in *P. pastoris* have been performed. It was reported that the highest heterologous production of chicken lysozyme was achieved in *P. pastoris*, with a protein concentration of 400 mg/L and antibacterial activity of 6.1 × 10^4^ U/mg in a 3-L fermenter cultivation (Masuda et al., 2005). In contrast, Wei et al. recently constructed a recombinant *P. pastoris* strain GShLY4-6 using G418 as a selection marker, in which the extracellular secreted hLYZ reached an antibacterial activity of 533 U/mL against *M. lysodeikticus* under shaking flask cultivation condition (Wei et al., 2012). Later, using the Plackett–Burman (PB) design and response surface methodology (RSM) methods, Yu et al. optimized the cultivation conditions to enhance the secretory levels of hLYZ in *P. pastoris* to present an antibacterial activity of 3,301 and 47,680 U/mL in shaking flask and 15-L fermenter high-density cultivation conditions, respectively, Yu et al. (2014).

Optimizing the gene codon according to the *P. pastoris* genetic preference and increasing the target gene copies in *P. pastoris* chromosome have been proven as two efficient methods for heterologous protein production in *P. pastoris* (Shu et al., 2016; He et al., 2019). Moreover, whether a protein can be well folded determines its secretory efficiency in *P. pastoris* (Idiris et al., 2010; Young and Robinson, 2014) because misfolded proteins in cell ER could induce an unfolded protein response (UPR) and endoplasmic reticulum-associated degradation (ERAD) (Ahmad et al., 2014). Like other eukaryotic cells, many molecular chaperons existed in the ER and Golgi of *P. pastoris* to help the proper folding of proteins (Juturu and Wu, 2018). For example, BiP, a major member of the Hsp70 chaperone family, binds to unfolded polypeptide chains and mediates protein folding within the ER (Rose et al., 1989), and ER-associated protein disulfide isomerase 1 (Pdi1) prompts the disulfide bond formation process (Farquhar et al., 1991), which is re-oxidized by endoplasmic thiol oxidase 1 (Ero1) for repetitive use (Hudson et al., 2015).

**FIGURE 1:**
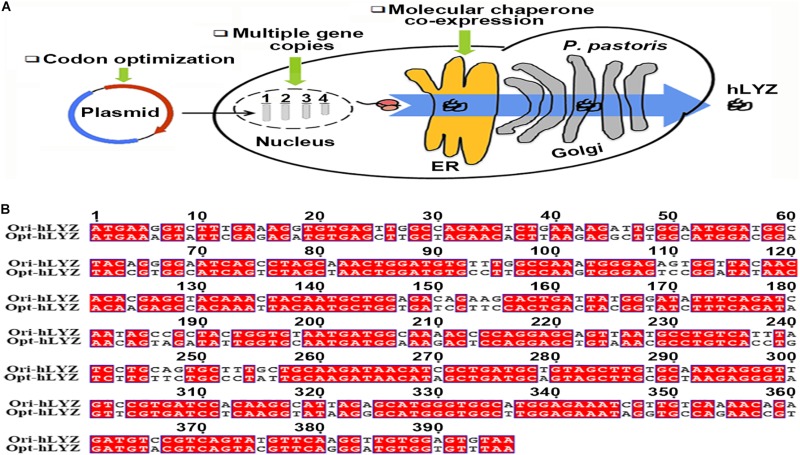
The heterologous expression strategy and codon-optimized hLYZ gene. **(A)** Schematic diagram for improving the heterologous production of human lysozyme expression in *Pichia pastoris.* **(B)** Nucleotide sequence alignment between the original and optimized human lysozyme gene.

In our studies here, the strategies of codon optimization, multiple gene copies, and molecular chaperone co-expression were integrated to prompt the transcriptional, translational, and post-translational efficiencies of hLYZ in *P. pastoris* GS115 (Figure 1A). Our results indicated that increasing the gene copy number of opt-hLYZ to six led to a highly improved mRNA transcription and protein production levels. Moreover, the tandem co-expression of Ero1 and Pdi1 together under GAP promotor further enhanced the extracellular secretion of hLYZ. Finally, the recombinant opt-hLYZ-6C-EP strain that had the highest production rate was applied to a 5-L high-cell-density cultivation, producing a total secreted hLYZ of 2.34 ± 0.02 mg/mL with an antibacterial activity against *M. lysodeikicus* of 1.76 ± 0.02 × 10^5^ U/mL. These results presented the highest production and activity of human lysozyme in *P. pastoris* that have ever been reported. Besides, the combinational strategy established in our studies may also guide the heterologous expression of other proteins in *P. pastoris*.

## Materials and Methods

### Strains, Plasmids, and Medium

*Pichia pastoris* GS115 was purchased from Invitrogen (Carlsbad, CA, United States), and *M. lysodeikticus* (CGMCC 1.4547) cells were purchased from China General Microbiological Culture Collection Center. The template plasmids p905M, pGAPZB, pGAPZM, and pGAPZN were generated in our laboratory. All the media, including the Luria–Bertani media (LB), Yeast Extract Peptone Dextrose media (YPD), buffered glycerol-complex media (BMGY), buffered methanol-complex media (BMMY), minimal dextrose media (MD), and Skerman’s basal mineral salt media (BSM) were prepared based on the Invitrogen PichiaPink^TM^ Expression System manual^1^.

Zeocin antibiotics, PageRuler^TM^ Prestained Protein Ladder and T4 DNA ligase were purchased from Thermo Fisher Scientific (Waltham, MA, United States). 5,5′-Dithiobis-2-nitrobenzoic acid (DTNB), N-acetyl-L-cysteine, Tris(2-carboxyethyl) phosphine hydrochloride (TCEP), and Hieff^TM^ PCR Master Mix were purchased from Yeasen (Shanghai, China). All other restriction enzymes were purchased from New England Biolab (Ipswick, MA, United States).

### Construction of Recombinant Expression Plasmids

Human lysozyme gene (GenBank accession number: M19045.13) was optimized based on the codon usage bias of *P. pastoris* from the website https://sg.idtdna.com/CodonOpt (Figure 1B). The optimized human lysozyme gene sequence (opt-hLYZ) was uploaded to the GenBank database (GenBank accession number: MN175974) and synthesized by Sangon Co., Ltd. (Shanghai, China). Then, the opt-hLYZ gene was inserted into the plasmid p905M between *Cpo*I/*Not*I restriction sites to construct the recombinant p905M-opt-hLYZ-1C plasmid based on a previously reported method (He et al., 2019). Subsequently, plasmids containing 3, 6, and 12 copies of opt-hLYZ (p905M-opt-hLYZ-3C, p905M-opt-hLYZ-6C, p905M-opt-hLYZ-12C) were constructed based on p905M-opt-hLYZ-1C plasmid using *Xba*I/*Bam*HI and *Spe*I/*Bam*HI restriction sites.

### Construction of Plasmids for Overexpression of the Chaperones

The DNA fragments of Bip (GenBank: AY965684.1), Ero1 (GenBank: AOA66382.1), and Pdi1 (GenBank: AOA70013.1) were amplified from *P. pastoris* GS115 genome, followed by insertion into the plasmid pGAPZB with restriction sites of *Eco*RI/*Xho*I to construct plasmids of pGAZB-Bip, pGAPZB-Ero1, and pGAPZB-Pdi1, respectively. At the same time, the Bip and Ero1 fragments were also inserted into the plasmid pGAPZM, and Ero1 and Pdi1 fragments were inserted into the pGAPZN using *Eco*RI/*Xho*I restriction sites, respectively, forming the pGAPZM-Bip, pGAPZM-Ero1, pGAPZN-Ero1, and pGAPZN-Pdi1 plasmids. Then, the tandem plasmids pGAPZM-Bip-(GAP^∗^)-Ero1, pGAPZM-Bip-(GAP^∗^)-Pdi1, and pGAPZM-Ero1-(GAP^∗^)-Pdi1 were constructed by cloning the expression cassettes between the restriction sites *Xba*I/*Bam*HI in the pGAPZN-Ero1 or pGAPZN-Pdi1 plasmids into the pGAPZM-Bip or pGAPZM-Ero1 plasmids, respectively, using the *Spe*I/*Bam*HI restriction sites.

### Yeast Transformation and Screening

The constructed p905M-opt-hLYZ-1C, p905M-opt-hLYZ-3C, p905M-opt-hLYZ-6C, and p905M-opt-hLYZ-12C plasmids were linearized by *Sal*I, followed by transformation into *P. pastoris* GS115 through electroporation (Life Technologies Cell Porator, Carlsbad, CA, United States), which generated the recombinant strains of opt-hLYZ-1C, opt-hLYZ-3C, opt-hLYZ-6C, and opt-hLYZ-12C, respectively. The positive transformants were selected on MD plates without histidine and confirmed by colony PCR. Subsequently, the plasmids containing different molecular chaperones, including pGAZB-Bip, pGAPZB-Ero1, pGAPZB-Pdi1, pGAPZM-Bip-(GAP^∗^)-Ero1, pGAPZM-Bip-(GAP^∗^)-Pdi1, and pGAPZM-Ero1-(GAP^∗^)-Pdi1, were linearized by *Avr*II and transformed into the opt-hLYZ-6C competent cells, generating recombinant strains of opt-hLYZ-6C-B, opt-hLYZ-6C-E, opt-hLYZ-6C-P, opt-hLYZ-6C-BE, opt-hLYZ-6C-BP, and opt-hLYZ-6C-EP, respectively. These strains were screened on YPDZ plates with Zeocin antibiotics (100 μg/mL).

### Yeast Total RNA Extraction and qRT-PCR Experiments

Cells were grown in the BMGY media till the OD_600_ reached approximately 15. Then, cells were induced by methanol for 24 h, followed by collection for mRNA quantification. Yeast total RNA was extracted from uninduced cells and induced cells, respectively, using the Yeast RNAiso kit (Takara Inc., Shiga, Japan), followed by reverse-transcription into cDNA in a 20-μL reaction mixture using the PrimeScript^TM^ RT Reagent kit with gDNA Eraser (Takara Inc., Shiga, Japan). The cDNA levels were then analyzed using the CFX real-time PCR system (Bio-Rad, Hercules, CA, United States). Relative expression levels against endogenous β-actin were determined with efficiency correction and associated technical errors on triplicates being calculated. Primers of actin-F (CCAATGAACCCAAAGTCCAA)/actin-R (CCGTCACCAGAGTCCAAAAC) and hLYZ-F (CCTGTCAC CTGTCTTGTTCTG)/hLYZ-R (CCCATGCCCTTATACCTTG AG) were used for β-actin and hLYZ quantification, respectively.

### Yeast Genomic DNA Extraction and PCR Amplification of hLYZ Expression Cassettes

The genomic DNA was extracted from recombinant opt-hLYZ-1C, -3C and -6C strains, respectively, Hoffman and Winston (1987). Primers of Genome-hLYZ-F (CTG ATCCTCATCAACTTGAGGGGCACTATC) and Genome- hLYZ-R (GCTCCGAGAACGGGTGCGACTAGAAATTGC) were used to amplify the hLYZ gene expression cassettes with the Hieff PCR^TM^ Master Mix. The PCR program was set as pre-denaturation (94°C/3 min), 30 cycles of denaturation (92°C/25 s)–annealing (56°C/25 s)–elongation (68°C/1.5–8 min), and final extension (68°C/10 min). The elongation time was set to 1.5, 3.5, and 8 min for genomic DNA amplification of the recombinant opt-hLYZ-1C, -3C, and -6C strains, respectively.

### Strain Cultivation in Shaking Flasks and 5-L Fermenter

The recombinant *P. pastoris* GS115 strains were inoculated into 50 mL of BMGY media and cultivated for 36 h at 28°C, 250 rpm. When the OD_600_ of the cells reached approximately 15, cells were harvested and re-suspended in 25 mL of BMMY media, followed by induction with 1% (v/v) methanol. The cultivated supernatants were collected every 24 h and assayed with SDS-PAGE.

For a 5-L fermenter high-cell-density cultivation, the recombinant opt-hLYZ-6C-EP cells was first inoculated into a flask containing 200 mL of YPD media and cultivated at 28°C, 250 rpm. When the OD_600_ reached to about 10, the cell cultures were transferred into a 5-L fermenter with 2 L of BSM media for high-cell-density cultivation (Baoxing, Shanghai, China). The glycerol was supplied as a carbon source for cell growth, followed by induction of methanol. During the cultivation, samples were collected and monitored for its OD_600_ and wet weigh every 12 h starting from methanol induction. The supernatant of the collected samples was obtained by centrifugation at 12,000 × *g* for 2 min. The yield of the secreted hLYZ was determined with protein concentration and assayed using SDS-PAGE.

### Determination of Disulfide Bonds in Purified hLYZ With Ellman’s Test

After methanol induction, the medium supernatant was first centrifuged at 4°C, 12,000 rpm for 10 min to remove yeast cells or insoluble impurity. The collective supernatant was filtrated with a 0.22-μm filter membrane and loaded onto the HiTrap SP FF prepacked column (GE, Boston, MA, United States). After washing with 20-mM Na_2_HPO_4_–NaH_2_PO_4_ buffer (pH 7.4), hLYZ was eluted using the same buffer containing 600-mM NaCl. Free sulfhydryl in the purified hLYZ was quantitated using the Ellman’s test with a calibration of N-acetyl-L-cysteine (Ellman, 1959; Bulaj et al., 1998). At the same time, the reduced hLYZ was prepared by incubating purified hLYZ with 8-mM TCEP, which was removed by PD-10 desalting column (Amersham biosciences, United Kingdom). Free sulfhydryl in the reduced hLYZ was quantitated again using the Ellman’s test.

### Determination of the Antibacterial Activity of hLYZ

The bacteriolytic activity of extracellular secreted hLYZ against *M. lysodeikticus* was determined according to the standard spectrophotometry method (GB/T 30990-2014). The medium supernatant from shaking flask and high-cell-density cultivation experiments was first collected and filtered to remove bacteria and residual yeast cells. For quantification of hLYZ antibacterial activity, *M. lysodeikticus* cells were first resuspended in pH 6.2, 100-mM phosphate buffer evenly to adjust the initial OD_450_ to approximately 1.3. After that, 1 mL of the cells was divided into a cuvette, and 50 μL of the diluted sample was added, followed by continuous recording of the cell optical density at 450 nm. The OD_450_ value difference between 15 and 75 s was used for calculating the antibacterial activity of hLYZ. The commercially bought human lysozyme (Cat. No.: L1667; Sigma) and the phosphate buffer were used as positive and negative controls, respectively.

## Results

### Secretory Production of hLYZ in Recombinant *P. pastoris* Strains With Multiple Gene Copies in Shaking Flask Cultivation Experiments

**FIGURE 2:**
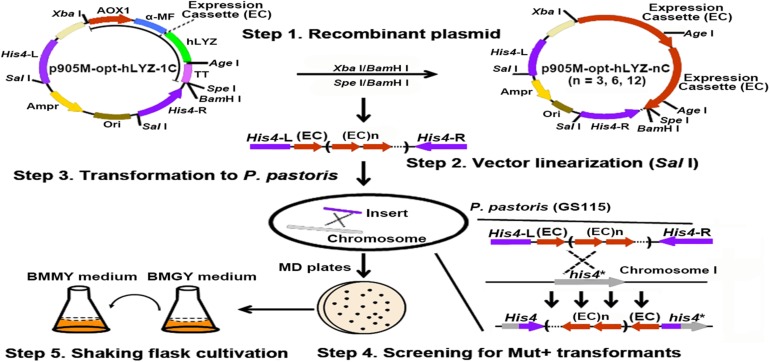
Strategy for construction of the recombinant *P. pastoris* strains with multiple gene copies of opt-hLYZ. Generating and characterizing the recombinant *P. pastoris* GS115 strains with multiple copies of opt-hLYZ gene included five major steps. The recombinant plasmid construction (Step 1) was followed by plasmid linearization using restriction enzyme *Sal*I (Step 2), yeast cell transformation (Step 3), recombinant strain identification (Step 4), and shaking flask cultivation (Step 5). The expression cassette (EC) in the plasmid opt-hLYZ-nC (*n* = 3, 6, 12) indicated the expression cassette of opt-hLYZ, and n indicated the number of expression cassette.

The human lysozyme gene has a length of 396 bp, encoding a 131-aa length of polypeptide with a calculated molecular mass of 14,831 Da^2^. In improving the heterologous protein production levels in *P. pastoris*, the most simple and efficient strategies are optimizing the target gene codons based on the genetic preference of *P. pastoris* and increasing its gene copies integrated into the *P. pastoris* genome. Normally, an antibiotic-resistant gene was co-expressed with the target gene, followed by the use of correspondent antibiotics, such as G418 for selecting recombinant strains. However, the expression of the antibiotic-resistant gene may increase the burden of *P. pastoris* host cells, thus, undermining the expression of the target hLYZ. Here, we used a gene-expression-cassette multimerization strategy to avoid introducing the antibiotic-resistant gene (Figure 2). First, the hLYZ gene (opt-hLYZ) was codon optimized based on the codon usage bias of *P. pastoris* (Figure 1B). Then, the opt-LYZ gene was inserted into the p905M plasmid by the T5 cloning method between the restriction sites of *Cpo*I and *Not*I, forming the p905M-opt-hLYZ-1C plasmid, which contains one copy of the opt-hLYZ gene (Xia et al., 2019). Subsequently, the plasmids p905M-opt-hLYZ-3C, p905M-opt-hLYZ-6C, and p905M-opt-hLYZ-12C, which contains 3, 6, and 12 copies of the opt-hLYZ genes, were constructed (Figure 2, Step 1). The constructed plasmids were then linearized by *Sal*I (Figure 2, Step 2), and transformed into *P. pastoris* GS115 by electroporation, respectively (Figure 2, Step 3). The opt-hLYZ expression cassettes were inserted into the *His4*/*his4* genes in the *P. pastoris* GS115 chromosome I through homologous recombination. Subsequently, the recombinant *P. pastoris* strains, including opt-hLYZ-1C, opt-hLYZ-3C, opt-hLYZ-6C, and opt-hLYZ-12C, which contain 1 to 12 copies of the opt-hLYZ expression cassettes were screened on MD plates without histidine and confirmed by yeast colony PCR (Figure 2, Step 4). The confirmed recombinant strains were finally examined for hLYZ production through shaking flask cultivation (Figure 2, Step 5).

**FIGURE 3:**
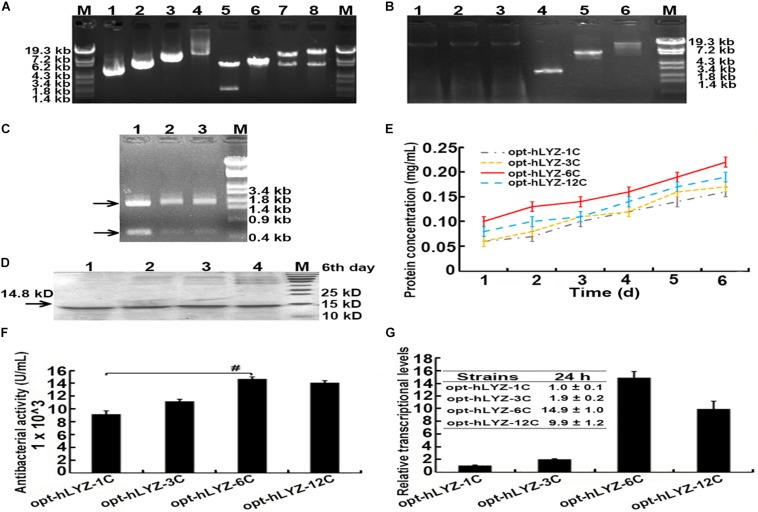
Characterization of the recombinant *P. pastoris* strains with multiple gene copies of hLYZ. **(A)** Confirmation of recombinant plasmids: Lanes 1/2/3/4: the plasmids p905M-opt-hLYZ-1C/3C/6C/12C, respectively; Lanes 5/6/7/8: the plasmids digested by the *Xba*I and *Bam*HI restriction enzymes. **(B)** Confirmation of genome-integrated hLYZ expression cassettes. Lanes 1/2/3: the extracted genome DNA of the recombinant opt-hLYZ-1C, 3C, and 6C strains, respectively; Lanes 4/5/6: the PCR amplification of the genome-integrated hLYZ expression cassettes of recombinant opt-hLYZ-1C, 3C, and 6C strains, respectively. **(C)** Enzymatic digestion of PCR amplification products from **(B)** using *Age*I. Lanes 1/2/3: against the recombinant opt-hLYZ-1C, 3C, and 6C strains, respectively. **(D)** SDS-PAGE analysis of extracellularly secreted hLYZ in medium supernatant. Lanes 1/2/3/4: the sample of recombinant opt-hLYZ-1C/3C/6C/12C strains, respectively, after 6 days of methanol induction. **(E)** Line chart of protein concentrations induced for 6 days of recombinant opt-hLYZ-1C/3C/6C/12C strains, respectively. **(F)** Antibacterial activity against *M. lysodeikicus* of extracellularly secreted hLYZ from recombinant opt-hLYZ-1C/3C/6C/12C strains. **(G)** The relative transcriptional levels of opt-hLYZ gene in recombinant opt-hLYZ-1C/3C/6C/12C strains, respectively. The data are presented as mean ± SEM (*n* = 3 independent experiments) with Student’s *t*-test being performed, ^#^*P* ≤ 0.05.

After obtaining the recombinant opt-hLYZ-1C/3C/6C/12C strains, the genome-integrated hLYZ gene copy numbers in these strains were validated. First, plasmids p905M-opt-hLYZ-1C/3C/6C/12C were digested with *Xba*I/*Bam*HI restriction enzymes to confirm the expression cassettes with lengths of 1,995, 5,985, 11,970, and 23,940 bp, respectively (Figure 3A, lanes 5–8). The length of the rest plasmid backbone is 6,140 bp, which is close to the length of expression cassette containing three hLYZ gene copies (Figure 3A, lane 6). Subsequently, PCR amplification of the genome-integrated hLYZ expression cassettes in these recombinant strains was performed to evaluate the gene copy numbers in the *P. pastoris* chromosome. The results showed the amplified DNA fragments with lengths of 2,556, 6,546, and 12,531 bp, corresponding to the one, three, and six copies of the hLYZ gene expression cassette, respectively (Figure 3B). Unfortunately, we failed to amplify the DNA fragment of the 12 copies of the hLYZ gene expression cassette after multiple attempts, as it is a very large fragment with the length of 23,943 bp (data not shown). These PCR products were further confirmed by digestion with the *Age*I restriction enzyme, which is located between the hLYZ gene and transcription terminator sequence in the expression cassette (Figure 2). Either two or three small fragments with lengths of 555, 1,995, and 2,001 bp, respectively, were produced (Figure 3C), further confirming the right expression cassette patterns of hLYZ in these recombinant strains.

The gene dosage effects of hLYZ in *P. pastoris* were then evaluated through shaking flask cultivation experiments (Figures 3D,E). Along with methanol induction, the secreted hLYZ protein in the media supernatant continuously increased. Our results showed that the recombinant opt-hLYZ-6C strain presented the highest amount of secretory hLYZ after the methanol induction of 6 days. Correspondingly, the media supernatant of the recombinant opt-hLYZ-6C strain also exhibited the highest antibacterial activity of 14,680 ± 300 U/mL against *M. lysodeikticus* (Figure 3E). For the recombinant opt-hLYZ-6C strain, the secretory hLYZ protein in the media supernatant increased from 0.10 ± 0.01 mg/mL on the first day of induction to a maximum of 0.22 ± 0.02 mg/mL after 6 days of induction. Comparably, the secreted hLYZ protein for the recombinant opt-hLYZ-1C, opt-hLYZ-3C, and opt-hLYZ-12C strains were 0.14 ± 0.02, 0.17 ± 0.01, and 0.19 ± 0.01 mg/mL, respectively, after 6 days of induction. The antibacterial activity against *M. lysodeikticus* of the recombinant opt-hLYZ-1C, opt-hLYZ-3C, and opt-hLYZ-12C strains were 9,200 ± 500, 11,200 ± 300, and 14,100 ± 300 U/mL, respectively (Figures 3D–F).

An interesting finding was that the secretory hLYZ production of the recombinant opt-hLYZ-12C strain, which contains 12 copies of the opt-hLYZ gene, was lower than that of the recombinant opt-hLYZ-6C strain, which only contains six copies of the opt-hLYZ gene. The mRNA levels of the hLYZ gene were also quantitated by qPCR in the recombinant opt-hLYZ-1C/3C/6C/12C strains (Figure 3G). After a 24-h methanol induction, compared to the transcriptional levels of the hLYZ gene in the recombinant opt-hLYZ-1C strain, the mRNA levels of the hLYZ gene increased to about 1.9-fold, 14.9-fold, and 9.9-fold in the recombinant opt-hLYZ-3C, opt-hLYZ-6C, and opt-hLYZ-12C strains, respectively, with the highest mRNA levels of hLYZ in the recombinant opt-hLYZ-6C strain.

### Secretory Expression of hLYZ in Recombinant *P. pastoris* Strains With Different Molecular Chaperones in Shaking Flask Cultivation Experiments

**FIGURE 4:**
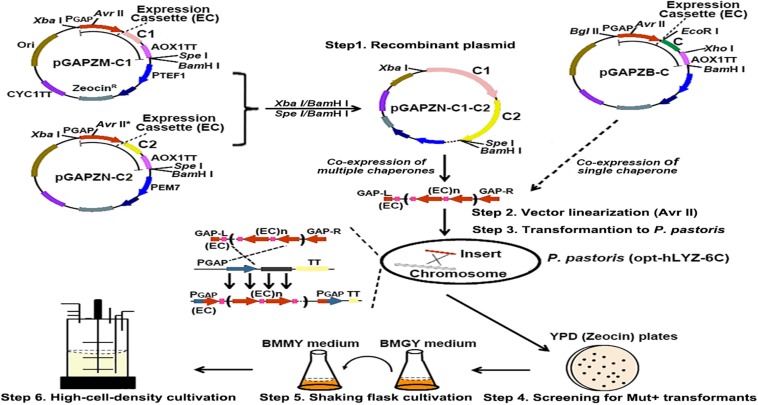
Strategy for construction of the recombinant *P. pastoris* strains with different molecular chaperones. The scheme of co-expression of different molecular chaperones in the recombinant opt-hLYZ-6C strain. Recombinant plasmid construction (Step 1) was followed by plasmid linearization using restriction enzyme *Avr*II (Step 2), yeast cell transformation (Step 3), recombinant strain identification (Step 4), shake flask cultivation (Step 5), and high-density cultivation (Step 6). The C, C1, and C2 in the plasmid pGAPZB, pGAPZM-C1, and pGAPZN-C2 indicated the molecular chaperones.

The human lysozyme has four intramolecular disulfide bonds, which play critical roles in maintaining its active confirmation (Taniyama et al., 1991). In eukaryotic cells, post-translational processes, such as protein folding and secretion, were mainly mediated through the ER and Golgi. To help in its folding and secretion in *P. pastoris*, we co-overexpressed three molecular chaperones, Bip, Ero1, and Pdi1, in the recombinant opt-hLYZ-6C strain due to their reported properties of prompting protein folding or disulfide bond formation. First, the Bip, Ero1, and Pdi1 gene fragments were amplified from the *P. pastoris* genome and then cloned into the pGAPZB plasmid between the *Eco*RI/*Xho*I restriction sites, forming the pGAPZB-Bip, pGAPZB-Ero1, and pGAPZB-Pdi1 plasmids (Figure 4). These constructs were subsequently linearized by *Avr*II and transformed into the recombinant opt-hLYZ-6C strain, generating the recombinant strains of opt-hLYZ-6C-B, opt-hLYZ-6C-E, and opt-hLYZ-6C-P for co-overexpression of Bip, Ero1, or Pdi1, respectively. In these recombinant strains, the expression cassettes of different molecular chaperones were inserted into the *PGAP*/*pgap* promoter region in the *P. pastoris* GS115 chromosome II through homologous recombination.

Additionally, the added-up effects of any two of these three molecular chaperons were also investigated in our studies. To facilitate the tandem expression of these molecular chaperones, two plasmids, the pGAPZM and pGAPZN plasmids, were constructed with minor modification of the pAGPZB plasmid (Figure 4). pGAPZM plasmid was constructed by introducing a *Spe*I restriction site close to the *Bam*HI in the pAGPZB plasmid and moving the *Xba*I restriction site outside the P_*GAP*_ promoter to facilitate the tandem gene cloning. Based on pGAPZM, the *Avr*II site was mutated to generate the pGAPZN plasmid. First, using the *Eco*RI/*Xho*I restriction sites, the Bip and Ero1 fragments were cloned into the pGAPZM plasmid, and the Ero1 and Pdi1 fragments were cloned into the pGAPZN plasmid, respectively, forming the pGAPZM-Bip, pGAPZM-Ero1, pGAPZN-Ero1, and pGAPZN-Pdi1 constructs. Then, the Ero1 and Pdi1 expression cassettes were cleaved off from the pGAPZN-Ero1 and pGAPZN-Pdi1 constructs using *Xba*I/*Bam*HI, respectively, which were cloned into the *Spe*I/*Bam*HI-designed pGAPZM-Bip and pGAPZM-Ero1 constructs to finally generate the pGAPZM-Bip-(GAP^∗^)-Ero1, pGAPZM-Bip-(GAP^∗^)-Pdi1, and pGAPZM-Ero1-(GAP^∗^)-Pdi1 plasmids (Figure 4, Step 1). Since *Xba*I and *Spe*I are isocaudomers, the *Xba*I/*Bam*HI-digested fragments from the pGAPZN plasmid could be cloned into the *Spe*I/*Bam*HI-digested pGAPZM plasmid with correct orientation. Besides, since the *Avr*II site in the pGAPZN plasmid was mutated, only the *Avr*II site in the pGAPZM was retained, which enabled the subsequent vector linearization of the final incorporated plasmid by *Avr*II. After linearization of the constructed pGAPZM-Bip-(GAP^∗^)-Pdi1, pGAPZM-Bip-(GAP^∗^)-Ero1, and pGAPZM-Ero1-(GAP^∗^)-Pdi1 plasmids by *Avr*II (Figure 4, Step 2), they were transformed into the recombinant opt-hLYZ-6C strain to generate the recombinant strains of opt-hLYZ-6C-BP, opt-hLYZ-6C-BE, and opt-hLYZ-6C-EP (Figure 4, Step 3). All these recombinant strains were then confirmed by yeast colony PCR (Figure 4, Step 4), followed by characterization in shaking flask cultivation experiments (Figure 4, Step 5). The best recombinant strain that gave the highest secretory hLYZ production was finally applied in the 5-L fermenter for the high-cell-density cultivation experiment (Figure 4, Step 6).

**FIGURE 5:**
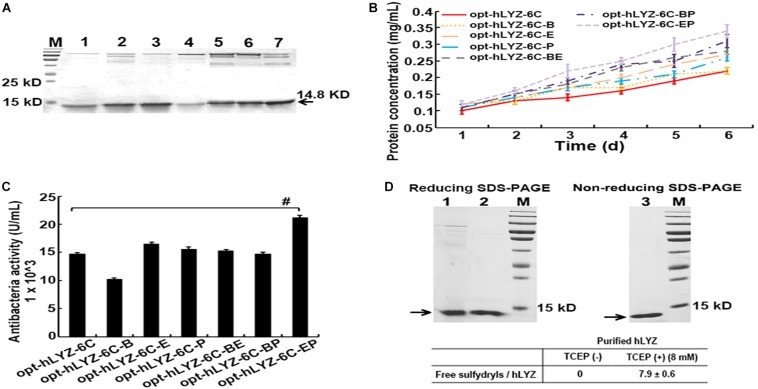
Characterization of the recombinant *P. pastoris* strains with different molecular chaperones. **(A)** SDS-PAGE analysis of extracellularly secreted hLYZ in medium supernatant. Lanes 1/2/3/4/5/6/7: The samples of recombinant opt-hLYZ-6C and opt-hLYZ-6C-Bip/Ero1/Pdi1/BE/BP/EP strains, respectively, after 6 days of methanol induction. **(B)** Line chart of extracellularly secreted hLYZ induced for 6 days of recombinant opt-hLYZ-6C and opt-hLYZ-6C-Bip/Ero1/Pdi1/BE/BP/EP strains, respectively. **(C)** Antibacterial activity against *M. lysodeikicus* of extracellularly secreted hLYZ in recombinant opt-hLYZ-6C and opt-hLYZ-6C-Bip/Ero1/Pdi1/BE/BP/EP strains, respectively. **(D)** Top: Lanes 1/2: hLYZ samples before and after purification with analysis of reducing SDS-PAGE analysis; Lane 3 purified hLYZ with analysis of non-reducing SDS-PAGE analysis. Bottom: quantitation of free sulfhydryl in purified hLYZ using Ellman’s test.

In the shaking flask cultivation experiments, our results showed that in the co-expression of either Pdi1 or Ero1 both increased the secretory production of hLYZ after 6 days of methanol induction, from 0.22 ± 0.02 to 0.26 ± 0.01 mg/mL and 0.27 ± 0.01 mg/mL, respectively (Figures 5A,B), with enhanced antibacterial activity against *M. lysodeikicus* of 15,600 ± 360 and 16,500 ± 300 U/mL (Figure 5C). It was noticed that although the co-expression of Bip did not change the secretory production of hLYZ (0.21 ± 0.01 mg/mL), the antibacterial activity of the secreted hLYZ decreased to 10,200 ± 280 U/mL. Besides, added-up effects of the Pdi1 and Ero1 molecular chaperones were observed. Simultaneously co-expressed Pdi1 and Ero1 in the recombinant opt-hLYZ-6C-EP strain presented the highest secretory production of hLYZ up to 0.34 ± 0.02 mg/mL with an antibacterial activity of 21,200 ± 300 U/mL against *M. lysodeikicus* (Figures 5A–C).

### Determination of Free Sulfhydryl in the Secreted hLYZ With Ellman’s Test

Four intramolecular disulfide bonds exist in hLYZ. To evaluate the homogeneous status of secreted hLYZ, secreted hLYZ was purified through a prepacked HiTrap SP FF cation exchange column, followed by the Ellman’s test (Ellman, 1959; Bulaj et al., 1998) to quantitate the free sulfhydryl (Figure 5D). The purified hLYZ presented a major single band with a molecular weight of approximately 15 kD in both the reducing and non-reducing SDS-PAGE (Figure 5D, top). Using N-acetyl-L-cysteine for calibration, no free sulfhydryl was detected in the purified hLYZ (Figure 5D, bottom). In comparison, approximately 7.9 ± 0.6 free sulfhydryls/hLYZ were detected in the TCEP-reduced hLYZ, corresponding to the eight cysteine residues in hLYZ. These results indicated that the secreted hLYZ existed in a monomer status with all cysteines in hLYZ being involved in disulfide bond formation.

### Heterologous Production of hLYZ in High-Cell-Density Cultivation

**FIGURE 6:**
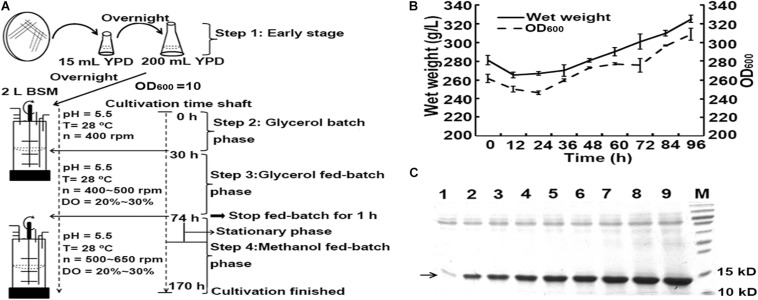
High-density cultivation of hLYZ in 5-L fermenter. The recombinant opt-hLYZ-6C-EP strain was further evaluated in 5-L high-density cultivation experiments. Every 12 h after methanol induction, 6 mL of each sample was taken from the fermenter and centrifuged, followed by SDS-PAGE assay with 10 μL of supernatant. **(A)** Optimized procedure of high-cell-density cultivation. **(B)** Growth curve of opt-hLYZ-6C-EP strain in the methanol fed-batch phase. **(C)** SDS-PAGE analysis of extracellular-secreted hLYZ in high-density cultivation experiments. Lanes 1–9: cultivation samples after 0 h/12 h/24 h/36 h/48 h/60 h/72 h/84 h/96 h of methanol induction, respectively. The data are presented as mean ± SEM (*n* = 3 independent experiments).

In the shaking flask cultivation experiments, the recombinant opt-hLYZ-6C-EP strain with six copies of the opt-hLYZ gene and co-expression of Pdi1 and Ero1 presented the highest hLYZ production as well as antibacterial activity (Figure 5A). Therefore, it was chosen for high-density cultivation in a 5-L fermenter. The cultivation procedure was optimized by maintaining the whole cultivation process at 28°C and pH 5.5, including glycerol batch phase, glycerol fed-batch phase, and methanol fed-batch phase (Figure 6A). In addition, 20% of dissolved oxygen (DO) was stably maintained in the fed-batch phase. Starting from around 74 h when OD_600_ reached 262, cells were induced with 100% methanol with PTM1 trace salts at a rate of 6 mL/h, starting from the methanol induction phase. After that, the cultivation entered into a stationary phase caused by methanol inhibition for approximately 48 h. In this stationary phase, it was critical to tightly control the supplement rate of methanol to minimize its toxicity to cells because switching the carbon source from glycerol to methanol might decline the amounts of mitochondrial respiratory chain and ATP production in *P. pastoris* GS115 (Li et al., 2019). Once passing this stationary phase, the cell biomass and OD_600_ started increasing until the cultivation procedure was finished after the methanol induction of 96 h. When the high-density cultivation procedure was finished, the final wet weight of the cells reached to 325 g/L, and the OD_600_ climbed up to 308.5 (Figure 6B). Besides, our results also showed that the amount of extracellular secreted hLYZ increased along with the cultivation process and finally reached 2.34 ± 0.02 mg/mL after methanol induction for 96 h (Figure 6C), which presented a bacteriolytic activity of 1.76 ± 0.02 × 10^5^ U/mL against *M. lysodeikicus*.

## Discussion

Lysozyme possesses a high economic value for human health and environmental protection on antibiotic replacement. However, the difficult large-scale production of human lysozyme hinders its practical application. Using a combinational strategy of gene codon optimization, multiple gene copies, and molecular chaperone co-expression, here, we constructed a robust recombinant *P. pastoris* GS115 strain, opt-hLYZ-6C-EP, in which the heterologous human lysozyme was extracellularly secreted with high antibacterial activity. The recombinant opt-hLYZ-6C-EP strain was further applied to the high-cell-density cultivation in a 5-L fermenter, finally producing the secretory hLYZ of 2.34 ± 0.02 g/L with an antibacterial activity against *M. lysodeikicus* of approximately 1.76 ± 0.02 × 10^^5^ U/mL, which is the highest that has ever been reported in *P. pastoris*.

As a typical yeast, *P. pastoris* possesses both microbial and eukaryotic properties. *P. pastoris* can be easily manipulated with fast growth rate, and it is also an ideal host for producing human proteins, such as hLYZ that contains four intramolecular disulfide bonds, due to its eukaryotic properties. Moreover, another two advantages of *P. pastoris* are that it has strong protein secretion ability useful for simplifying subsequent protein purification process and its high-density cultivation procedure was well developed for industrial settings (Chung et al., 2013). Many strategies have been characterized in improving the secretory production of protein in *P. pastoris*. First, codon optimization is an effective strategy, which could minimize the influence of codon bias against the heterologous gene in host cells, thus prompting its translational efficiency. It has been reported that proper codon usage could balance the optimal and non-optimal codons to help fine tune mRNA levels, thus prompting translation efficiency (Lanza et al., 2014; Presnyak et al., 2015; Ahn et al., 2016; Zhou et al., 2016; Hitzeroth et al., 2018). Through codon analysis (Figure 1B), ACG or CGC rare codons that are mainly responsible for Ser or Arg were replaced in the original human lysozyme gene sequence.

Gene multimerization is a direct way to increase the mRNA levels of the heterologous gene through integrating multiple gene copies into the *P. pastoris*’s chromosome. In here, 3, 6, and 12 copies of the opt-hLYZ gene were integrated into the *P. pastoris*’s chromosome, respectively, all of which exhibited an elevated hLYZ expression (Figure 3). It has to be pointed out that the highest hLYZ expression was observed in the recombinant opt-hLYZ-6C strain instead of the recombinant opt-hLYZ-12C strain (Figure 3D). The subsequent RT-PCR experiments suggested that the mRNA levels of hLYZ in opt-hLYZ-6C was the highest, which was approximately 0.5-fold higher than that of opt-hLYZ-12C (Figure 3G). Although more gene copies theoretically will lead to higher mRNA levels, there is still no clear explanation for this non-corresponding result. It only can be speculated that transcription is a complex procedure, which is regulated by many factors and varies in different cells (Vogel and Marcotte, 2012).

Many studies have suggested that protein secretion in yeast cells is not only determined by its expression levels but also depends on its folding status (Hou et al., 2012; Young and Robinson, 2014). The human lysozyme has four intra-molecular disulfide bonds, which might bring difficulties for its correct folding in *P. pastoris*. Therefore, co-expressing three molecular chaperones, Bip, Pdi1, and Ero1, to enhance the post-translational efficiency was applied in our studies. These three molecular chaperones were reported to function on prompting on protein folding and disulfide bond formation (Rose et al., 1989; Farquhar et al., 1991; Hudson et al., 2015) and applied to successfully increase the heterologous expression of hydrophobin HFBI (Sallada et al., 2019), *Candida rugosa* lipase Lip1 (Li et al., 2016), and human albumin (HSA) fusion protein IL2–HSA in *P. pastoris* (Guan et al., 2016). In our studies here, Pdi1 and Ero1 exhibited positive effects on the secretory expression of hLYZ in *P. pastoris* (Figure 5A). More interestingly, Pdi1 and Ero1 presented added-up effects wherein co-overexpressing Pdi1 and Ero1 together will further enhance the secretory expression of hLYZ from 0.22 to 0.34 mg/mL, with an antibacterial activity of 21,200 ± 300 U/mL against *M. lysodeikicus*. Since Pdi1 and Ero1 are both mainly involved in introducing disulfides into target proteins in the ER (Oka and Bulleid, 2013), and it has been reported that disruption of the disulfide bonds in hLYZ might lower its expression and catalytic activity (Taniyama et al., 1988; Omura et al., 1991), we speculated that disulfide bond formation in the post-translational process is one of the bottlenecks for effective production of hLYZ in *P. pastoris*. In fact, no free sulfhydryl was detected in the purified hLYZ from the recombinant opt-hLYZ-6C-EP strain (Figure 5D, bottom), and purified hLYZ presented a major single band (14.8 kD) in both reducing and non-reducing SDS-PAGE (Figure 5D, top), indicating that the secretory hLYZ is a monomer and fully oxidized. However, it was also noticed that co-expression of Bip decreased the antibacterial activity of secretory hLYZ for approximately 30% (Figure 5C). The combinational function of molecular chaperones in ER is still elusive (Nishikawa et al., 2005), thus, how to design a combinational of multiple molecular chaperones to improve the heterologous production of a target protein in *P. pastoris* is still under exploration.

Different from the conventional method of selecting recombinant strains with multiple genes integrated using antibiotics, such as Zeocin and G418, we adopted the *in vitro* gene-expression-cassette multimerization method previously reported by us to assemble multiple hLYZ expression cassettes (He et al., 2019). Using this method, we assembled up to 12 gene expression cassettes and integrated them into the *P. pastoris* genome (Figure 2). More importantly, this method could avoid the potential side effects introduced by the antibiotic-resistant gene and significantly simplified the subsequent selection process of recombinant strains. In addition, using a similar strategy, we could easily facilitate a tandem expression of the different molecular chaperones in *P. pastoris* (Figure 4).

To test its potential for large-scale industrial production, high-density cultivation procedure was preliminarily investigated in our studies (Figure 6). Our studies showed that pH control was of great importance in the high-density cultivation of the human lysozyme. When the pH was adjusted to 6.0 in the methanol fed-batch phase, the cultivation did not perform well with a very low amount of extracellularly secreted hLYZ. In contrast, keeping the pH of 5.5 and proceeding with the whole cultivation process at 28°C significantly increased the secretory hLYZ. In addition, strict control of the methanol feeding rate is also crucial in the stationary phase. It could be speculated that when the *P. pastoris* cells are in the process of adapting to the carbon change, its cellular metabolism slows down and will be more vulnerable to the toxicity of methanol. However, after the stationary phase, *P. pastoris* cells will have adapted to methanol, so it will prompt cell growth and protein production.

The combinational strategy developed in our studies significantly increased the hLYZ production in *P. pastoris* through synergistically targeting the enhancing of its transcriptional, translational, and post-translational efficiencies. Besides, our preliminary exploration of its high-density cultivation procedure also helps its potential large-scale production in the industry. Considering that protein expression and secretion is a complex process, this strategy could be applied as a general guide for other heterologous protein productions in *P. pastoris*.

## Data Availability Statement

All datasets generated for this study are included in the article/supplementary material.

## Author Contributions

HH, SW, MM, JN, and CL performed the experiments. HH and LY wrote the manuscript and designed the experiments. LM and GZ were involved in the analysis and interpretation of the experimental data. LY conceived the idea and supervised the whole research. All authors read and approved the final version of the manuscript.

## Conflict of Interest

The authors declare that the research was conducted in the absence of any commercial or financial relationships that could be construed as a potential conflict of interest.

## Footnotes

1 www.thermofisher.com

2 www.expasy.org
